# Emergency departments are part of the maternal health solution: Findings from piloting an educational toolkit in Illinois

**DOI:** 10.1002/aet2.70046

**Published:** 2025-05-08

**Authors:** Katherine A. Craemer, Lauren Sayah, Emilie Glass‐Riveros, Cara J. Bergo, Autumn Mels, Roma Allen, Diedra Stewart, Stacie E. Geller

**Affiliations:** ^1^ Center for Research on Women and Gender, College of Medicine University of Illinois Chicago Chicago Illinois USA; ^2^ Illinois Department of Public Health Office of Women's Health and Family Services Chicago Illinois USA; ^3^ University of Chicago Medicine Perinatal Center Chicago Illinois USA; ^4^ Loyola University Medical Center Perinatal Center Maywood Illinois USA; ^5^ South Central Illinois Perinatal Center, St. John's Children's Hospital Springfield Illinois USA; ^6^ Department of Obstetrics and Gynecology, College of Medicine University of Illinois Chicago Chicago Illinois USA

**Keywords:** education, emergency medicine, maternal health, mental health, substance use disorders

## Abstract

**Background:**

In Illinois, an average of 88 pregnant and postpartum individuals died annually from 2018 to 2020. Of these, 66% had at least one emergency department (ED) visit. To improve maternal health outcomes, the Illinois Maternal Mortality Review Committees recommended ED education on obstetric care, mental health conditions, and substance use disorders (SUD).

**Methods:**

From January 1, 2023, to June 30, 2024, the Maternal Health Emergency Department Toolkit (Toolkit) training was piloted in six diverse Illinois hospitals. The Toolkit's effectiveness was evaluated using electronic medical record (EMR) data to assess changes in practice within the ED.

**Results:**

A total of 281 (63%) ED providers and staff completed the training. From pre‐ to post‐pilot, EMR documentation of pregnancy or postpartum status in female patients of reproductive age increased from 56.4% to 83.4%, and screening pregnant and postpartum patients for mental health or SUD rose from 52.2% to 69.6%.

**Conclusions:**

The Toolkit is an evidence‐based training of educational modules that led to positive changes in ED provider and staff's practice when caring for pregnant and postpartum patients. By fostering education, engagement, and collaboration, the Toolkit has the potential to play a key role in helping reduce maternal mortality and morbidity in Illinois. The Toolkit is now available statewide for all Illinois EDs.

## INTRODUCTION

In the United States from 2010 to 2020, 8.6% of all emergency department (ED) visits were female patients aged 15–44 years old. Additionally, 3% of all ED visits were pregnant patients, equating to 2.77 million pregnant patients visiting the ED annually.^1^ While there is limited research available on ED use among cases of severe maternal morbidity (SMM) and maternal mortality, available research demonstrates that pregnant and postpartum patients who experience these adverse outcomes are often visiting EDs. A study in Massachusetts identified that high ED use during pregnancy was associated with an increased risk of SMM and a significant proportion of pregnant patients visited multiple EDs.^2^ In Illinois, from 2018 to 2020, an average of 88 people died per year during pregnancy or within 1 year postpartum.^3^ Of these pregnancy‐related deaths, 91% were identified as potentially preventable by the Illinois Department of Public Health Maternal Mortality Review Committees (MMRCs).^3^ Notably, 66% of those who died while pregnant or within 1 year postpartum visited the ED at least once during this period, averaging three or more visits.^3^ Additionally, 90% of rural individuals who died while pregnant or within 1 year postpartum accessed the ED at least once during this time.^3^ These findings suggest that perinatal patients are utilizing ED services and we should explore opportunities to improve maternal health outcomes through ED engagement and education.

To improve maternal health outcomes, many national organizations have recommended education and training to support EDs and ensure that they are prepared to care for pregnant and postpartum patients.^4^, ^5^ In November 2024, the Centers for Medicare & Medicaid Services (CMS) released a new standard requiring all emergency services to have adequate provisions and protocols to meet the needs of obstetric (OB) patients and for all ED staff to complete this training every 2 years.^6^ Following case reviews of maternal deaths in Illinois, the MMRCs recommended enhanced education and engagement in OB care, mental health, and substance use disorders (SUDs) for all Illinois EDs.^3^, ^7^

In Illinois, maternal health education and support for EDs is limited. When ED administrators, educators, and health care providers search for maternal health education, the materials on providing care for pregnant and postpartum patients in the ED are insufficient and rarely informed by ED experts.^8^ In response to the recommendations made by the Illinois MMRCs, a task force of 33 multidisciplinary experts on maternal health and emergency medicine created the Maternal Health Emergency Department Toolkit (Toolkit) training.^8^ The Toolkit training contains five educational modules, consisting of didactic information, case‐based learning, and resources for additional reading and local implementation. The modules focus on data from the Illinois MMRCs, triage and management of emergencies in perinatal patients, screening and treatment of mental health and SUDs, addressing trauma during pregnancy, performing resuscitation during pregnancy, and conducting safe and coordinated discharge of perinatal patients from the ED.

Providing education and resources to ED clinicians and staff that address the gaps identified by the Illinois MMRCs may be an opportunity to improve maternal health outcomes. We piloted the Toolkit training to evaluate its effectiveness. This paper outlines how the Toolkit impacted provider and staff's practice caring for pregnant and postpartum patients in the ED at these pilot hospitals.

## METHODS

### Study design

From January 1, 2023, to June 30, 2024, the Toolkit training was piloted at six hospitals in Illinois. The pilot program was led by the University of Illinois Chicago (UIC) Center for Research on Women and Gender within the College of Medicine and the Administrators and Obstetric Educators at the University of Chicago, South Central and Loyola regional administrative perinatal centers (APC). The online, self‐guided 3.75‐hour training consisting of five learning modules was offered to all nurses, physicians, advanced practice providers, and staff working in the pilot hospital EDs. The training was primarily hosted on the Qualtrics survey platform (Version June 2024) where each module had a designated survey with embedded training video and applicable evaluation questions. Participants were assigned a random number that they entered to track completion. When available, the training was also offered through the hospital's learning management system and through attending live webinars. Completion data were exported from the Qualtrics surveys, the learning management systems, and webinar attendance sheets. Following completion of all training modules and the pre‐ and post‐training surveys, participants received their choice of three continuing medical education or continuing education credits.

### Key outcome measures

The success of the pilot program was assessed by the project outcome measures of: (1) displaying a sign in the ED alerting patients to inform ED staff of their pregnancy or postpartum status; (2) asking female patients of reproductive age (10–55 years old) if they are pregnant or within 1 year postpartum; (3) documenting the patient's pregnancy or postpartum status in the patient's health record; and (4) screening, referring, and treating patients who are pregnant or within 1 year postpartum in accordance with the guidelines taught in the Toolkit. Selection to capture patients 10–55 years old was made by the task force who developed the Toolkit. This range was selected based on experience caring for perinatal patients to ensure all potential patients from onset of menses to menopause were captured.

### Setting and population

The hospitals identified to participate in the pilot were selected based on a variety of geographic locations, racial and ethnic groups of patient population, and perinatal acuity level. The pilot hospitals were located across Illinois: one was in Chicago, three were in the suburbs of Chicago, and two were in central Illinois. Two pilot hospitals served predominately non‐Hispanic (NH)‐white communities; one hospital served a predominately NH‐Black community; one hospital served primarily Hispanic and NH‐white communities; one hospital served primarily Hispanic and NH‐Black communities; and one hospital served primarily NH‐Black, NH‐white, and Hispanic communities.^9^ Categorized by the perinatal regionalization system,^10^ the pilot hospitals included two nonbirthing hospitals without OB units, one Level I hospital that provided care to low‐risk pregnant persons and newborns, two Level II hospitals that provided care to moderate‐risk pregnant persons and newborns, and one Level III hospital that provided care to high‐risk pregnant persons and operated a neonatal intensive care unit for newborns.

### Sign in the ED

The presence of a sign in the ED alerting patients to disclose to ED staff that they are pregnant or have been pregnant in the past year was assessed at the beginning and end of the pilot. The presence of a sign was reported through email correspondence during the first quarter of the pilot and photographic evidence of a sign in the ED was emailed to the research team during the final quarter of the pilot program.

### Electronic medical record (EMR) data analysis

EMR data captured changes in practice to assess whether ED clinicians asked female patients of reproductive age (10–55 years old) if they were pregnant or within 1 year postpartum, whether the ED clinicians recorded the patient's pregnancy or postpartum status in the patient's health record, and whether ED clinicians treated pregnant and postpartum patients in accordance with the guidelines taught in the Toolkit. Guidelines included referring or transferring pregnant and postpartum patients to a higher level of care, communicating with an OB or maternal fetal medicine (MFM) provider before discharging pregnant and within 12 weeks postpartum patients from the ED, and screening all pregnant and postpartum patients not experiencing a life‐threatening emergency for mental health conditions and SUDs.

Using a standardized abstraction questionnaire, each pilot hospital extracted EMR data five times during the pilot program: at baseline prior to the training being offered and every quarter (*n* = 4) during the pilot. The first four data extractions included 3 months of data, and the fifth data extraction included 2 months of data. Due to small samples sizes, the fourth and fifth data extractions were combined to create a final data set for analysis. Additionally, due to changes in data collection across the project, assessment of mental health and SUD screenings began at the second data extraction and only five of the six pilot hospitals extracted the related data.

The extracted data were a random sample of 10% of female patients of reproductive age per month, for a minimum of 10 patients per month. If the minimum was not met, hospitals were asked to oversample until they reached 10 patients per month. Data were extracted from checkboxes and free‐texted data within a patient's medical record. EMR data were collected through Qualtrics software (Version June 2024) and Microsoft Excel spreadsheets (Version 2808). Aggregate data were analyzed though descriptive statistics and chi‐square analysis using SPSS (Version 29.0.2.0). Analysis included baseline versus final data from all pilot hospitals and subanalyses of between and within birthing and nonbirthing hospitals and between and within rural and nonrural hospitals. This study was designated as exempt from review by the UIC Institutional Review Board.

## RESULTS

### Characteristics of study subjects

Of the 445 ED providers and staff at the pilot hospitals, a total of 281 (63.1%) ED employees completed the training and 397 (89.2%) finished at least one training module. Of the ED employees who finished the Toolkit, nurses (*n* = 155, 55.2%) and physicians (*n* = 81, 28.8%) were the two roles with the highest representation (Table 1).

**TABLE 1:** Employees who started and employees who completed the Toolkit by employee role.

Role	Started	Finished
Nurse	198 (49.9)	155 (55.2)
Physician	122 (30.7)	81 (28.8)
Physician assistant	11 (2.8)	6 (2.1)
Administrative	10 (2.5)	7 (2.5)
Social worker	1 (0.3)	0 (0.0)
Nurse practitioner	4 (1.0)	2 (0.7)
Uknown	6 (1.5)	3 (1.1)
Other	45 (11.3)	27 (9.6)
Total	397 (100.0)	281 (100.0)

*Note*: Data are reported as *n* (column %).

Abbreviation: Toolkit, Maternal Health Emergency Department Toolkit.

### Sign in the ED

From pre‐ to post‐pilot, the reported presence of a sign in the ED alerting patients to inform ED staff of their pregnancy or postpartum status increased from 0% to 100%.

### EMR data

A total of 1764 records for female patients of reproductive age were extracted during the pilot program. Of these patients, 0.2% were Asian, 33.4% were Black/African American, 10.3% were Hispanic/Latina, 49.8% were white, 2.0% had more than one race/ethnicity listed, and 4.3% did not have their race/ethnicity listed (data not shown).

**FIGURE 1:**
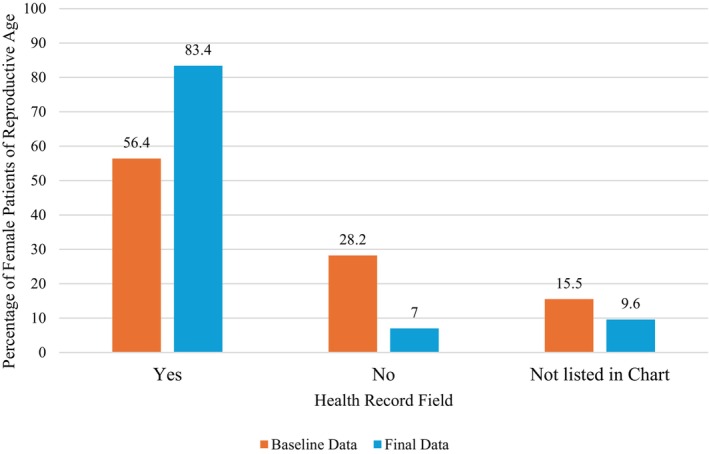
EMR documentation of asking female patients of reproductive age if they are pregnant or postpartum, aggregate baseline and final data. EMR, electronic medical record.

From baseline to final data extraction, the percentage of all patients of reproductive age who were asked if they were pregnant or postpartum and had this status documented in the EMR significantly increased from 56.4% to 83.4% (*p* < 0.001; Figure 1). Practice changes were further indicated by the increase from 15.0% to 61.9% of patients that had a documented “no” to being pregnant or postpartum and a decrease from 43.6% to 18.1% of patient's pregnancy status not listed in chart. Of the 1764 extracted records, 318 (18.0%) patients were pregnant, and 36 (2.0%) patients were up to 1 year postpartum (data not shown).

Analysis of EMR data for pregnant and postpartum patients demonstrated a significant increase in the percentage of patients with a documented referral or transfer to a higher level of care from 43.2% at baseline to 56.8% at the final data extraction (*p* < 0.001; Table 2). Referring patients to an OB or MFM was the most common referral, and this increased from 81.2% to 87.3% but did not reach statistical significance (*p* = 0.110). Of patients who were pregnant or postpartum, the percentage with documented communication between an ED and an OB or MFM provider before the patient was discharged significantly increased from 30.1% to 56.4% (*p* < 0.001; Table 2). Of the pregnant and postpartum patients not experiencing a life‐threatening emergency, the percentage who received a mental health or SUD screening significantly increased from 52.2% to 69.6% (*p* < 0.008; Table 2).

**TABLE 2:** EMR documentation for treating pregnant or postpartum patients in accordance with the Toolkit guidelines, aggregate baseline and final data.

	Baseline data	Final data	*p*‐value
Referral or transfer to higher level of care documented			
Yes	32 (43.2)	79 (56.8)	
No	25 (33.8)	42 (30.2)	<0.001
Not listed in chart	2 (2.7)	13 (9.4)	
No referral or follow‐up needed	15 (20.3)	5 (3.6)	
Referral type			
OB or maternal fetal	25 (78.1)	55 (69.6)	
Medicine (MFM)			0.110
Other^a^	6 (18.8)	10 (12.7)	
More than one location^b^	1 (3.1)	14 (17.7)	
ED provider communicated with OB or MFM provider			
Yes	22 (30.1)	75 (56.4)	
No	29 (39.7)	28 (21.2)	
Not listed in chart	3 (4.1)	4 (3.0)	
Hospital does not collect information	11 (15.1)	0 (0.0)	<0.001
Other/notes about hospital procedure	8 (11.0)	26 (19.5)	
Received mental health or SUD screening			
Yes	24 (52.2)	64 (69.6)	
No	3 (6.5)	12 (13.0)	<0.008
Unknown	19 (41.3)	16 (17.4)	

*Note*: Data are reported as *n* (%).

Abbreviations: EMR, electronic medical record; MFM, maternal fetal medicine; OB, obstetric; SUD, substance use disorder; Toolkit, Maternal Health Emergency Department Toolkit.

^a^ Other referrals included: the patient died during transfer, mental health rehabilitation centers, the intensive care unit, and another hospital with higher level of care.

^b^ Referrals to more than one location included: OB, MFM, and administrative perinatal center.

The analysis between and within birthing and nonbirthing hospitals demonstrated that both birthing and nonbirthing hospitals benefited from the Toolkit, with birthing hospitals benefiting more (*p* < 0.0001, data not shown). The analysis between and within rural and nonrural hospitals demonstrated that both rural and nonrural hospitals benefited from the Toolkit, with nonrural hospitals benefiting more (*p* < 0.0001, data not shown).

## DISCUSSION

Maternal mortality is the tip of the iceberg in the larger context of pregnancy‐related complications that can cause long‐lasting health problems.^3^ Every year in the United States, more than 60,000 women experience life‐threatening SMM, resulting in more than 700 pregnancy‐related deaths annually.^11^ Available research has found that those who experience maternal mortality and SMM are often visiting EDs and many national organizations have recommended education and training to better support EDs caring for pregnant and postpartum patients.^2^, ^3^, ^4^, ^5^

Several national organizations offer training programs that include education and simulations to prepare non‐OB health care centers for labor, childbirth, and the management of birth complications. However, these programs primarily focus on physiological emergent OB conditions.^12^, ^13^, ^14^ For instance, Florida has developed an ED postpartum education package, which encompasses components such as assessing patients' postpartum status; recommending OB consultation; and providing education on mental health conditions, SUDs, emergent OB conditions, and discharge planning. However, this package is limited to the postpartum period.^15^ More recently, additional maternal education materials for EDs have been developed, but none comprehensively address the educational gaps that Illinois EDs need to effectively care for pregnant and postpartum patients. For example, in 2024, the American College of Obstetricians and Gynecologists introduced treatment algorithms for cardiovascular disease, acute hypertension, and eclampsia in non‐OB settings, intended for ED, emergency medical services, and urgent care practitioners.^16^ Additionally, the Alliance for Innovation on Maternal Health released the Obstetric Emergency Readiness Resource Kit, which includes simulations and preparations for hypertension, cardiac conditions, sepsis, SUDs, and mental health conditions to support preparedness efforts for non‐OB health care teams managing OB emergencies.^17^ While theses trainings are important for better supporting EDs, they do not fulfill the gaps identified by the Illinois MMRCs to meet the needs of EDs caring for pregnant and postpartum patients.

The Toolkit was developed to fill these gaps, and this study demonstrated the training as effective. The pilot program achieved its project outcome measures: (1) pilot hospitals had a sign in the ED alerting patients to inform the ED staff of their pregnant or postpartum status; (2) the percentage of patients of reproductive age asked if they were pregnant or within 1 year postpartum significantly increased; (3) documentation of the patient's pregnancy or postpartum status significantly increased; and (4) practice changes for treating patients who are pregnant or within 1 year postpartum in accordance with the guidelines taught in the Toolkit significantly improved. Practice changes included improved documentation of referrals and transfers to higher levels of care, augmented communication between an ED and OB or MFM provider before the patient was discharged, and increased percentage of pregnant and postpartum patients receiving a mental health or SUD screening. Practice changes may be the result of hospitals inducting new policies and practices, or ED employees increasing their awareness of existing policies and practices for pregnant and postpartum patients. These improvements not only indicate practice changes but suggest an overall culture change of caring for pregnant and postpartum patients.

With the continual closures of birthing and nonbirthing hospitals in Illinois and across the United States, it is increasingly critical to prepare EDs, especially those at nonbirthing hospitals and rural hospitals, to care for pregnant and postpartum patients.^2^, ^18^ Although providing OB care is not the primary role of the ED, pregnancy and postpartum persons are often visiting the ED for care.^3^ The Toolkit was designed to provide essential information to support the delivery of high‐quality care for pregnant and postpartum patients. Our pilot program intentionally included EDs without OB units and those in rural areas to assess whether the training was effective in these settings. The results of the pilot program found meaningful improvements in practice at EDs regardless of the presence of an OB unit or geographic location. Further, we captured an increase in documented communication between ED and OB or MFM providers indicating a merging of the commonly siloed OB and ED units. We recognize the important need to provide support and resources to areas with limited or no access to perinatal and other key health services, such as mental and behavioral health. While the Toolkit cannot rectify this lack of services, it provides EDs with key resources available in Illinois and includes critical information on caring for pregnant and postpartum patients when they come through the ED's doors.

## LIMITATIONS

The Illinois pilot program was limited by the small sample size of six hospitals included in the pilot. The results of the comparisons between the birthing versus nonbirthing, and the rural versus nonrural hospitals are also limited in their generalizability due to small sample sizes. Despite a small hospital sample size, the individual hospitals contributed a substantial number of patient records that were sufficient for aggregate analysis. The EMR data collection was also limited due to the 10% sample size. While this percentage is low, it was selected to ensure that data collection was feasible for hospitals to extract. Data collection on mental health and SUD screening may also have limited the results as they did not begin until the second data extraction due to a delay in confirming that the data was captured in patient's medical records and subsequent delay in adding the data field to the standardized abstraction questionnaire. Further, only five of the six pilot hospitals contributed to this data field because the medical records of one hospital did not capture mental health nor SUD screening data. Participation in the pilot may also have been limited by how the education was completed. Completion of the training modules was primarily hosted through individual survey links that required entering a randomly assigned number to identify the participant. The utilization of this system may have decreased initial and continued engagement as many participants may have forgotten their assigned number or found the process too cumbersome to engage. Overall, the completion of the Toolkit's educational modules was voluntary; therefore, the results may have a bias toward individuals who wanted to make changes to their practice.

Despite these limitations, 63.1% of ED employees completed the training and we found significant improvements in practice. These included increased documentation of pregnancy or postpartum status in the EMR, increased screening of pregnant and postpartum patients for mental health or SUD, and augmented communication between ED and OB or MFM providers.

## CONCLUSIONS

The Maternal Health Emergency Department Toolkit fills the gaps in education identified by the Illinois Maternal Mortality Review Committees and national organizations. We captured meaningful increases in documentation of pregnancy or postpartum status in female patients of reproductive age, improved screening of pregnant and postpartum patients for mental health or substance use disorders, augmented referrals to an obstetric or maternal fetal medicine provider, and increased communication between an ED and an obstetric or maternal fetal medicine provider before the patient was discharged. Overall, the results of this pilot program demonstrated that the Maternal Health Emergency Department Toolkit is an evidence‐based training that promoted positive changes in practice and knowledge regardless of the geographic location or presence of obstetric unit.

This proof‐of‐concept study demonstrated the feasibility of implementing a maternal health training in the ED setting and the impact of said training on culture change and practices in an ED. Based on findings from the pilot, the leadership team is currently implementing the Maternal Health Emergency Department Toolkit at all hospitals with EDs in Illinois. The knowledge learned from the Toolkit is already standard practice at the six pilot hospitals and is available for institutions across the United States to implement at their facilities. Overall, the Maternal Health Emergency Department Toolkit is an evidence‐based training of educational modules that has the potential to improve care for pregnant and postpartum people visiting EDs. We recommend implementing the Maternal Health Emergency Department Toolkit or similar training to all EDs across the United States.

## AUTHOR CONTRIBUTIONS

Katherine Craemer: Conceptualization; methodology; investigation; visualization; project administration; writing—original draft. Lauren Sayah: Methodology; data curation; investigation; formal analysis; writing—review and editing. Emilie Glass‐Riveros: Investigation; methodology; writing—review & editing. Cara Bergo: writing—review and editing. Autumn Mels: Investigation; writing—review & editing. Roma Allen: Investigation; writing—review and editing. Diedra Stewart: Investigation; writing—review and editing. Stacie Geller: Conceptualization; methodology; supervision; funding acquisition; project administration; writing—review and editing.

## FUNDING INFORMATION

This work was supported by the Illinois Department of Public Health Maternal and Child Health Title V Block Grant, work order #36300007K.

## CONFLICT OF INTEREST STATEMENT

The authors declare no conflicts of interest.

## Data Availability

The data that support the findings of this study are available from the corresponding author upon reasonable request.
